# Pre- and Neonatal Exposure to Lipopolysaccharide or the Enteric Metabolite, Propionic Acid, Alters Development and Behavior in Adolescent Rats in a Sexually Dimorphic Manner

**DOI:** 10.1371/journal.pone.0087072

**Published:** 2014-01-22

**Authors:** Kelly A. Foley, Klaus-Peter Ossenkopp, Martin Kavaliers, Derrick F. MacFabe

**Affiliations:** 1 Graduate Program in Neuroscience, The University of Western Ontario, London, Ontario, Canada; 2 Department of Psychology, The University of Western Ontario, London, Ontario, Canada; 3 The Kilee Patchell-Evans Autism Research Group, Department of Psychology, The University of Western Ontario, London, Ontario, Canada; 4 The Kilee Patchell-Evans Autism Research Group, Division of Developmental Disabilities, Departments of Psychology and Psychiatry, The University of Western Ontario, London, Ontario, Canada; Chiba University Center for Forensic Mental Health, Japan

## Abstract

Alterations in the composition of the gut microbiome and/or immune system function may have a role in the development of autism spectrum disorders (ASD). The current study examined the effects of prenatal and early life administration of lipopolysaccharide (LPS), a bacterial mimetic, and the short chain fatty acid, propionic acid (PPA), a metabolic fermentation product of enteric bacteria, on developmental milestones, locomotor activity, and anxiety-like behavior in adolescent male and female offspring. Pregnant Long-Evans rats were subcutaneously injected once a day with PPA (500 mg/kg) on gestation days G12–16, LPS (50 µg/kg) on G15–16, or vehicle control on G12–16 or G15–16. Male and female offspring were injected with PPA (500 mg/kg) or vehicle twice a day, every second day from postnatal days (P) 10–18. Physical milestones and reflexes were monitored in early life with prenatal PPA and LPS inducing delays in eye opening. Locomotor activity and anxiety were assessed in adolescence (P40–42) in the elevated plus maze (EPM) and open-field. Prenatal and postnatal treatments altered behavior in a sex-specific manner. Prenatal PPA decreased time spent in the centre of the open-field in males and females while prenatal and postnatal PPA increased anxiety behavior on the EPM in female rats. Prenatal LPS did not significantly influence those behaviors. Evidence for the double hit hypothesis was seen as females receiving a double hit of PPA (prenatal and postnatal) displayed increased repetitive behavior in the open-field. These results provide evidence for the hypothesis that by-products of enteric bacteria metabolism such as PPA may contribute to ASD, altering development and behavior in adolescent rats similar to that observed in ASD and other neurodevelopmental disorders.

## Introduction

Autism spectrum disorders (ASD) are neurodevelopmental disorders with roughly 4 males diagnosed for every 1 female. ASD comprise a number of behavioral symptoms, including impairments in communication, social behavior, sensory abnormalities, and restricted and repetitive behavior [1]. In many children and adults with ASD, psychiatric disorders, gastrointestinal symptoms and epilepsy comorbidly occur [2], [3].

It is becoming well established that both genetics and environmental factors contribute to the development and expression of ASD. A number of genes involved in immune function, mitochondrial function, and neural circuit formation have been implicated [4]. However, known genetic factors discovered thus far account for 10–20% of ASD and concordance rates among monozygotic twins are less than 100%, suggesting an important role for environmental risk factors which act on the underlying genetic susceptibilities [5].

The gastrointestinal tract (GI) is home to over a trillion commensal bacteria, known as the microbiome, that have a bidirectional relationship with the central nervous system and contribute to normal immune system development and homeostasis in both humans and rodents. GI dysbiosis has been implicated in inflammatory diseases and neuropsychiatric health [6], [7]. There is suggestive evidence that imbalances in the composition of the microbiome may also contribute to the development or maintenance of ASD in children with findings of abnormal levels of bacteria flora, including *Clostridia, Bacteroidetes*, and *Desulfovibrio* in the GI tract of autistic children. Many of these bacteria are antibiotic-resistant. As such, repeated early infections in postnatal life treated with antibiotics may provide an enteric environment that promotes overgrowth of these bacteria resulting in intestinal inflammation [8], [9].

Byproducts of these enteric bacteria (from carbohydrate and some protein metabolism) include the short chain fatty acid, propionic acid (PPA) [10], which are able to enter circulation and may alter immune function and/or exacerbate ASD behaviors. Although PPA is necessary for normal immune and physiological functioning, elevated levels may result in disruptive effects [6]. In fact, propionic acidemia is a neurodevelopmental metabolic disorder characterized by elevated levels of PPA that clinically resembles some aspects of autism [11]. A case study of autism occurring comorbidly with propionic acidemia has been reported [12] while elevated fecal levels of SCFA have been found in ASD children [13]. In adult male rats, central administration of PPA has produced hyperactivity, perseveration and decreased social behavior [14]. High levels of SCFA in the hindgut of rats and peripheral PPA injections have also produced changes in activity, anxiety-like, and social behavior, consistent with ASD [15], [16]. Neuroinflammatory and metabolic changes, implicating oxidative stress and mitochondrial dysfunction, have been observed in a subset of patients with ASD and in rats given central PPA [17], [18], [19].

Immune dysfunction may increase the risk for ASD with alterations in the adaptive and innate cellular immune responses having been observed in children (see [20] for review). Maternal immune activation (MIA) may be induced in rodents using poly I:C (a viral mimetic) or lipopolysaccharide (LPS, a bacterial mimetic) to investigate the role of the immune system in the development of anxiety, schizophrenia and ASD. Valproate (VPA), a common epilepsy treatment, has been shown to increase the risk of ASD. MIA and prenatal administration of VPA produces developmental delay and behavioral deficits in rodents [21], [22]. Brusque et al. administered daily PPA throughout postnatal life (days 6–28 of life) and reported developmental delay and motor impairment [23]. Interestingly, VPA shares some structural and pharmacological properties with PPA [14]. However, to date, there have been no studies examining the effects of prenatal PPA administration on behavior in either male or female offspring.

Prenatal and postnatal administration of immune stimulants (such as LPS) has shown to result in changes in anxiety-like and exploratory behavior in adult and adolescent male and female rats. Increased anxiety in the elevated plus maze have been shown in male and female adolescent and adult offspring [24], [25], [26]. Assessment of open-field activity has yielded mixed results, with decreased nose-hole pokes [27] and decreased activity and centre entries [28], no change in locomotor activity [27], [29], or increased activity [30] being observed. While there is increasing attention on investigating possible sex differences following MIA (e.g., [30]), most published studies, to date, are with male rodents.

Results of a number of studies have also shown that the effects of postnatal LPS on subsequent behavior do not manifest themselves unless a second environmental insult (e.g., restraint stress or LPS injection) is experienced in adulthood [26], [31]. Postnatal immune activation confers a susceptibility to later systemic insults that result in abnormal behavior. This idea, termed the double hit hypothesis, was put forward to describe the genetic predisposition in schizophrenia that may confer vulnerability to an environmental trigger later in life that results in emergence of the disorder [32]. Genetics may also confer a susceptibility to prenatal or postnatal environmental insults in ASD, or it may be that more than one insult may be required as is the case in repeated infections in early life. Acute or repeated immune responses may alter the composition of the gut microbiome, increasing production of potential aversive metabolic products [33], [34]. Thus, it is possible that prenatal treatment with PPA may leave offspring vulnerable to the effects of postnatal PPA treatment. Prenatal LPS may also leave offspring vulnerable to postnatal PPA, as LPS is also a product of enteric bacteria. This approach of multiple environmental insults has not been used in animal models of ASD thus far.

The present study investigated the effects of prenatal treatment with the immune stimulant, LPS, and the microbiome associated gastrointestinal factor, PPA, on postnatal developmental milestones, open-field activity and anxiety-like behavior in adolescent male and female offspring. In addition, the effects of a second ‘hit’ of PPA in the second postnatal week were examined. It was hypothesized that prenatal LPS would increase anxiety-like behavior in offspring and that prenatal PPA would increase locomotor activity and anxiety-like behavior in offspring. Combinations of prenatal and postnatal treatments, prenatal LPS with postnatal PPA and prenatal PPA with postnatal PPA, were included to assess whether behavioral effects were magnified compared to that seen after either treatment alone. Developmental delay was observed in male and female rats with prenatal PPA and LPS, while increased anxiety-like behavior was sex- and treatment-specific.

## Methods

### Ethics Statement

Procedures were approved by the University of Western Ontario Animal Care Sub-Committee and were in accordance with the Canadian Council of Animal Care (CCAC) guidelines (Protocol Number: 2008-063).

### Animals

Twelve primiparous female Long-Evans rats weighing between 270–310 g were mated with adult male Long-Evans rats (375–550 g, Charles River, Canada) for a total of 12 litters. Females were paired overnight with a male the night before behavioral estrus. Sperm present on a vaginal smear (hematoxylin & eosin stain) the morning after pairing indicated successful mating and this was designated gestational day 0 (G0). Dams were housed individually in standard polypropylene cages (45×22×20 cm) with *ad libitum* access to both food (ProLab RMH 3000) and water. A 12∶12 h light:dark cycle (lights on at 0700 h) was maintained in a temperature controlled colony room (21±2°C). Litters were born on G22 (designated as postnatal day (P) 0), toe-clipped for identification, and were weaned at P21 (*M* = 14.17 pups, *SD* = 2.41). On P21, pups were weaned and randomly culled to a maximum of 10 animals per litter (5 males, 5 females). Weaned rats were housed in same-sex, same-postnatal treatment groups of 2 or 3 in standard polypropylene cages under the same conditions as dams. All behavioral testing took place during the light phase and animals were monitored (e.g., body weight) during testing.

### Prenatal LPS and PPA Administration

Sodium propionate (PPA, P1880, Sigma Chemical, St. Louis, MO, USA) was dissolved in 0.1 M phosphate buffered saline and administered at a dose of 500 mg/kg (0.26 M) subcutaneously (SC, pH corrected to 7.4 with concentrated HCl) once a day on G12–16 for a total of 5 injections. This dose was selected based on previous studies and is meant to resemble states of metabolic dysfunction, thus a larger dose of PPA than that found in normal human colon was used (20 mM, [16], [35]). Injections started on G12 to mimic the VPA and MIA models of ASD [36]. Multiple injections were administered given the short half-life of PPA (20 min) [23]. Lipopolysaccharide (LPS from *E. coli* serotype 0111:B4, L2630, Sigma Chemical, St. Louis, MO, USA) was dissolved in 0.1 M phosphate buffered saline and administered SC at a low dose of 50 µg/kg on G15 and G16 for a total of 2 injections. Prenatal LPS administered at this time has been previously shown to increase anxiety-like behavior [24]. A low dose of LPS (compared to [24]) was used in order to be comparable to the relatively low dose of elevated PPA that may result from an altered microbiome composition [37], [38]. An equivalent volume of phosphate buffered saline was injected SC as a vehicle control (2 mL/kg) to yield two control groups, either 2 injections of VEH on G15 and G16 (2VEH) or 5 injections of VEH on G12–16 (5VEH). All maternal injections were administered between the shoulder blades.

### Postnatal PPA Administration

As synaptogenesis occurs during the first 3 weeks of postnatal life in rats [39], on P10, 12, 14, 16, and 18, male and female pups were injected twice a day SC with either PPA (500 mg/kg, pH corrected to 7.4 with concentrated HCl) or equivalent volumes of phosphate buffered saline vehicle (VEH, 5mL/kg) to correspond with an environmental insult in early human neonatal life. Half of each litter was injected with postnatal PPA and the rest with VEH. Injections took place at 0930 h (between the shoulder blades) and 1530 h (between the haunches).

### Experimental Procedure

**Figure 1:**
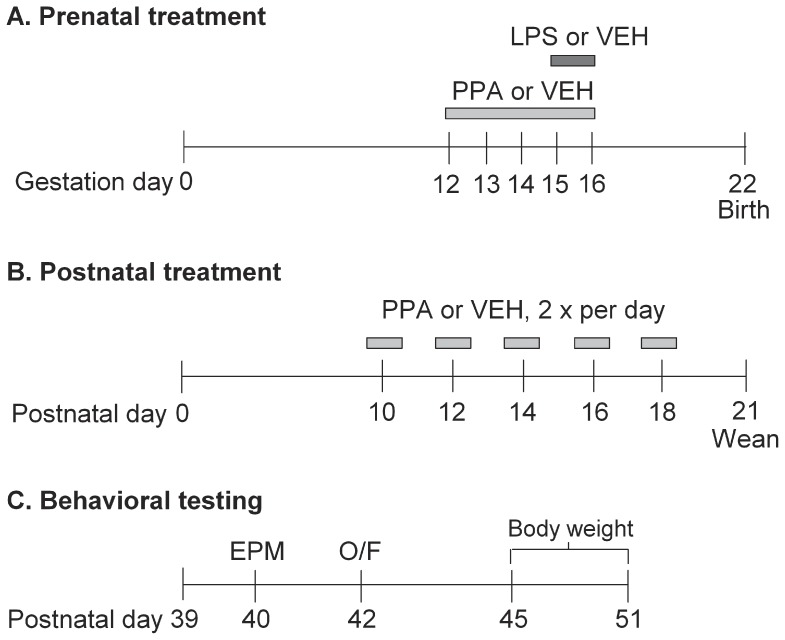
Study timeline. A: Prenatal treatment – Pregnant dams received propionic acid (PPA) or vehicle once a day from gestation day 12–16 to yield the prenatal PPA and 5VEH groups. Lipopolysaccharide or vehicle was administered once a day on gestation days 15 and 16 to yield the prenatal LPS and 2VEH groups. B: Postnatal treatment – Half the pups in all litters received PPA every second day (2 times a day) from P10–18 with the other half receiving vehicle. C: Behavioral testing in adolescence – EPM: elevated plus maze; O/F: open-field activity and thigmotaxis; behavioral data collected from P45–51 reported elsewhere.

A timeline showing drug administration and behavioral testing is provided in Figure 1. All pups were monitored for developmental milestones, with Table 1 providing a summary of treatments and group numbers of pups. On P21, litters were weaned to a maximum of 10 animals and these animals underwent behavioral testing in adolescence (Table 1). The prenatal and postnatal injection schedule yielded the following treatment combinations for each sex: No pharmacological treatment except vehicle (prenatal 2VEH or 5VEH with postnatal VEH); Prenatal treatment alone (prenatal LPS or PPA with postnatal VEH); Postnatal PPA alone (prenatal 2VEH or 5VEH with postnatal PPA); Prenatal and Postnatal treatment combined (prenatal LPS or PPA with postnatal PPA).

**Table 1:** Number of animals in the treatment groups used for developmental milestones and behavioral testing.

Sex	Postnatal treatment		Prenatal treatment			
		2VEH	LPS	5VEH	PPA	Total
Male	VEH	8 (6)	8 (6)	8 (6)	12 (6)	36 (24)
	PPA	10 (8)	8 (8)	11 (9)	14 (9)	43 (34)
**Total Males**		18 (14)	16 (14)	19 (15)	26 (15)	79 (58)
Female	VEH	9 (6)	12 (6)	12 (6)	10 (6)	43 (24)
	PPA	11 (8)	14 (9)	12 (9)	11 (9)	48 (35)
**Total Females**		20 (14)	26 (15)	24 (15)	21 (15)	91 (59)

Note: Numbers in the table represent number of animals per group for males and females. The first number indicates the number of pups assessed for developmental milestones, and the number in brackets indicates the number of animals that underwent behavioral testing. There were 3 litters in each of the 4 prenatal groups (2VEH, 5VEH: 2 or 5 injections of phosphate buffered saline vehicle on G15–16 or G12–16, respectively; LPS: Lipopolysaccharide, 50 ug/kg on G15–16; PPA: Propionic acid, 500 mg/kg on G12–16). Postnatal treatment during the second week of rat pups’ life consisted of phosphate buffered saline vehicle (VEH) or propionic acid (PPA). A maximum of 5 males and 5 females (3 postnatal PPA, 2 postnatal VEH for each sex) per litter were included in behavioral testing. Testing took place on P40 and 42.

#### Developmental milestones

The body weights of pups were monitored daily for the first 20 days of life. The following developmental milestones were assessed for day of appearance: Righting reflex (P2–6): pups were placed on their back and given 30 s to turn onto stomach with all limbs outstretched from body; Pinna detachment (P2–4): bilateral pinna unfolding completely from head; Incisor eruption (P7–13): both upper and lower; Eye opening (P12–16): scored as 0 =  both eyes closed, 1 =  one eye open, 2 =  both eyes open; Negative geotaxis (P7–10): time (s) to rotate 180° on a 30° incline when placed head down (assesses vestibular function and motor development) [36] with each pup given a maximum of 3–60 s trials to complete the task; Free-fall righting reflex (P15): pups were held 35 cm above a padded surface with back facing down and released. A successful trial occurred when the pup landed on its stomach, all limbs were outstretched with 3 successive trials (15 s apart) yielding a possible maximum score of 3.

#### Elevated plus maze (EPM) – P40

The EPM was made of wood and painted grey with non-toxic paint. The apparatus consisted of two opposite open arms (54×12 cm) with no sides or ends and orthogonal to two enclosed arms with sides and ends (54×12×48 cm). The four arms extended from a centre platform (12×12 cm) and the apparatus was raised 50 cm from the floor. An overhead camera connected to a television and DVD-R recorded behavior for later scoring.

Testing took place on the afternoon of P40. Animals were recorded for 5 minutes and placed on the centre platform facing an open arm to begin the test. After each animal, the maze was cleaned with a 20% alcohol solution. Measures assessed included the number of entries onto open and closed arms and time spent (s) on open and closed arms. Percent time in open arms was taken as a measure of anxiety (time spent in open arms/time spent in open arms+time spent in closed arms×100).

#### Open-field test – P42

Locomotor activity was monitored using eight Versamax Animal Activity Monitors (AccuScan Model DCM-8, Columbus, OH, USA), each consisting of a Plexiglas open field chamber (40 cm×40 cm×30.5 cm), and a Plexiglas lid with air holes. Infrared beams surrounding each chamber recorded horizontal and vertical locomotor activity as beam breaks, from which locomotor measures were compiled [40]. There were 16 infrared beam sensors on each side (2.54 cm apart, 4.5 cm from the floor) for horizontal movements, while on two opposite sides, 16 upper beams were located 15 cm above the chamber floor to assess vertical movements. Additionally, the VersaMax software separated the open-field into discrete periphery (7.5 cm wide border) and centre (30×30 cm square) zones to measure thigmotaxis (tendency of animals to stay close to the walls, an indication of anxiety).

Animals were placed in the novel open-field for 60 min on P42 to assess any changes in locomotor activity. Horizontal activity measures analyzed were: total distance (TD) − total horizontal distance (cm); horizontal movement time (MT) − amount of time (s) an animal was engaged in horizontal movement; number of horizontal movements (NM) − number of horizontal movements separated by 1 s stop time. Vertical activity measures analyzed were: vertical movement time (VT) − amount of time (s) an animal spent in a vertical position; number of vertical movements (VM) − number of vertical movements separated by 1 s stop time. Repetitive activity was measured using number of revolutions (clockwise and counterclockwise) - number of times an animal runs in a clockwise or counterclockwise circle of at least 2 inches in diameter. Duration spent (s) in the periphery and centre was measured, and locomotor activity was corrected for time spent in each zone (TD, MT, VM, VT).

### Statistical Analysis

All analyses were performed with IBM Statistics 20 (formerly Statistical Package for the Social Sciences, SPSS). As pups within a litter are not independent samples, the effects associated with belonging to a litter and being raised in a litter must be accounted for. To do this, linear mixed models were used for each of the dependent variables, with Litter used as a subject variable. Fixed factors in all models were: Sex, Prenatal drug, Postnatal drug, with litter size used as a covariate. For body weight, negative geotaxis, and eye opening, Day was also included as a factor. LSD post-hocs were performed. Significance was set to α = 0.05.

## Results

### Development

A Chi-square test was performed to verify that litter sizes did not significantly differ across groups, χ^2^(11) = 4.49, *p*>.05. There were also no significant differences in the number of male to female pups in each of the prenatal treatment groups, *F*(3,8) = 1.88, *p* = 0.211.

#### Body weight: postnatal

**Figure 2:**
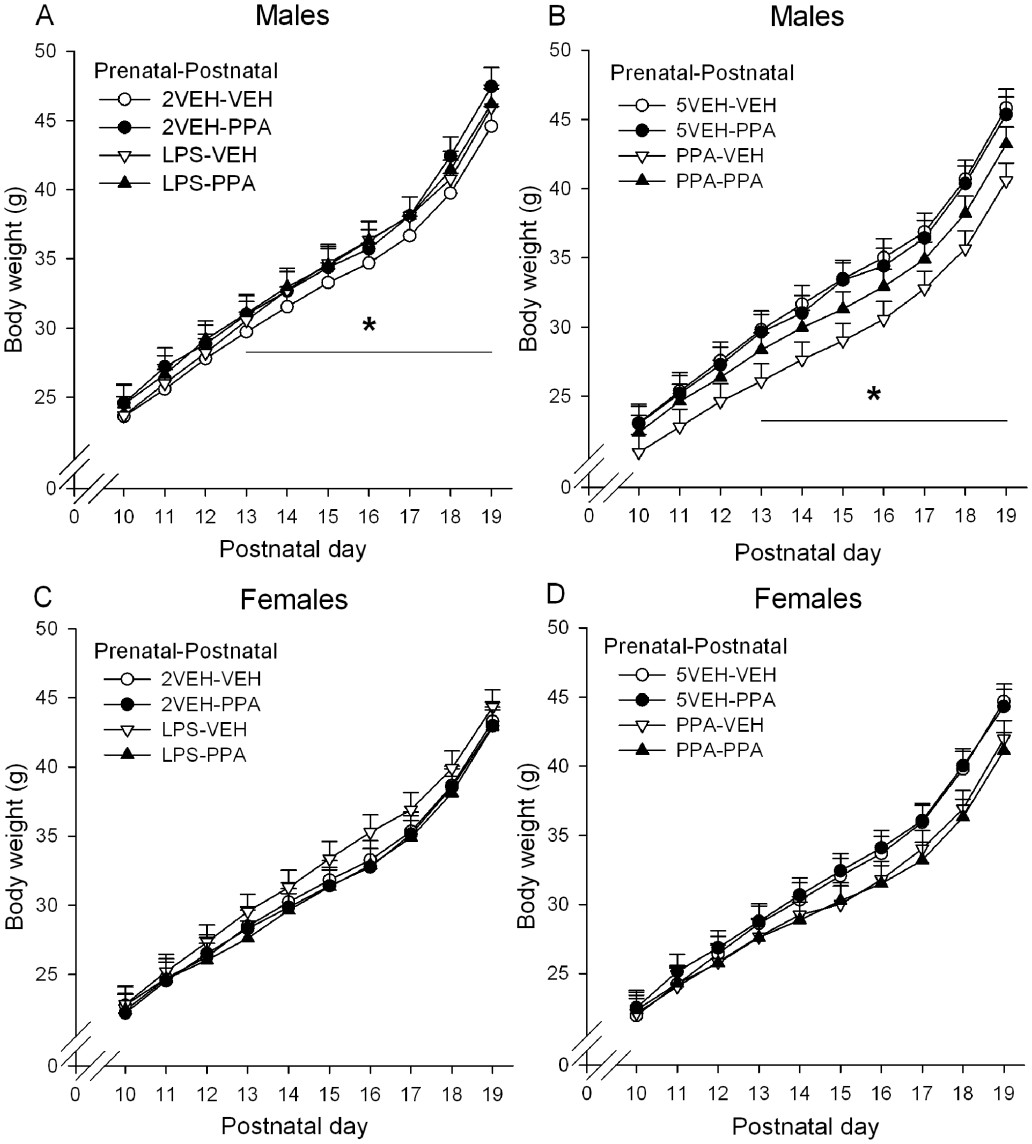
Body weight (g) for male and female offspring from postnatal days 10–19. A–B Males. C–D Females. Rats were prenatally exposed to either lipopolysaccharide (LPS) on G15–16, propionic acid (PPA) on G12–16, or their respective phosphate buffered saline controls (2VEH and 5VEH). Postnatal drug treatment, either PPA or VEH, was administered 2x/day every other day from P10–18. Males receiving postnatal PPA weighed significantly more than postnatal VEH treated males in the prenatal 2VEH (2VEH-VEH vs. 2VEH-PPA in Panel 1A) and prenatal PPA groups (PPA-VEH vs. PPA-PPA in Panel 1B), *p*s <0.05. Females receiving postnatal PPA weighed significantly less than postnatal VEH treated females (general effect, *p*<0.05). Error bars represent S.E.M. Refer to Table 1 for group designations and sample sizes.

Weight was monitored daily for the first 20 days of life (P0–P19). All pups gained weight across days, *F*(19,3072) = 3077, *p*<0.001, and male pups weighed significantly more than female pups, *F*(1,3072) = 88.02, *p*<0.001 (Figure 2A–D). There was a significant Sex×Prenatal drug×Postnatal drug interaction, *F*(3,3072) = 6.20, *p*<0.001. Birth weight and weight over the first 12 days of life did not significantly differ among prenatal treatments (Figure S1). Postnatal PPA treated male pups were significantly heavier than postnatal VEH treated male pups (*p*<0.001) on P13–19 in the prenatal PPA and 2VEH groups, *p*s <0.05 (Figure 2A–B). Postnatal PPA treated female pups were significantly lighter than postnatal VEH treated female pups (*p*<0.001) with individual days failing to reach significance in the prenatal LPS group (Figure 2C–D).

#### Body weight: adolescence

Weight was monitored on P39, P45, 47, 49, and 51 (behavioral data from P45–51 presented elsewhere). All animals gained weight across days, *F*(4,419) = 298.04, *p*<0.001, with males weighing significantly more than females, *F*(1,417) = 1841.06, *p*<0.001. A significant Sex×Prenatal drug×Postnatal drug interaction, *F*(3,417) = 3.36, *p* = 0.019, indicated that in prenatal 2VEH animals, postnatal PPA adolescent males weighed significantly more than postnatal VEH males, *p*<0.001 (P39 NS, P45–51 *p*s <0.05), with no significant effect of postnatal drug in adolescent females (Figure S2).

#### Physical milestones

**Figure 3:**
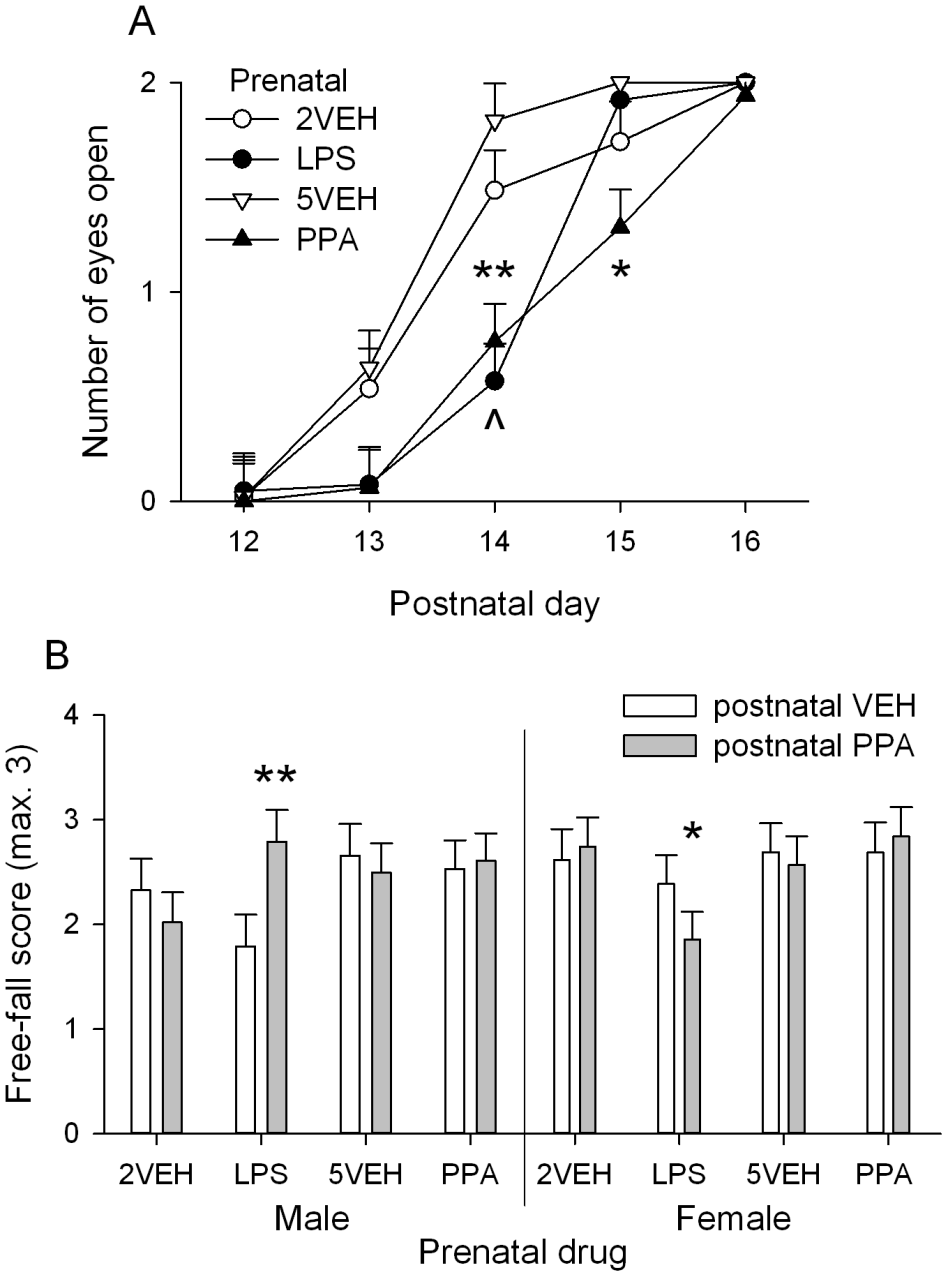
Developmental milestones for male and female offspring. A: Eye opening across postnatal days. Data is collapsed across sex and postnatal drug as there were no significant effects of these on eye opening. On P14, both prenatal LPS ( ^∧^, *p*<0.05) and PPA treated animals were delayed compared to vehicle treated controls. On P15, prenatal PPA treated pups continued to exhibit delayed eye opening. B: Free-fall righting reflex: 3 trials occurred, with a successful trial given a score of 1. In prenatal LPS treated pups, postnatal PPA produced sex differences, with higher scores in postnatal PPA treated males and lower scores in postnatal PPA treated females compared to postnatal VEH treated males and females, respectively. Error bars represent S.E.M. Refer to Table 1 for group designations and sample sizes. * *p*<0.05, ** *p*<0.01.

There was no developmental delay and no sex differences in pups as a result of either prenatal PPA or LPS treatment for eruption of top and bottom incisors, and pinna detachment (Figure S3A–C). There was developmental delay observed in day of eye opening in both prenatal PPA and prenatal LPS treated male and female pups (Figure 3A). A significant Day×Prenatal drug interaction, *F*(12,762) = 13.0, *p*<0.001, indicated that prenatal PPA and LPS treated male and female pups were significantly different from both 5VEH (*p*s <0.01) and 2VEH treated pups (*p*s <0.05) on P14, while on P15, prenatal PPA treated male and female pups were significantly different from 5VEH and LPS (*p*s <0.05).

#### Reflexes

The day at which rat pups could perform a righting reflex was monitored. A sex difference was present, *F*(1,147) = 6.70, *p* = 0.011, with males performing the reflex significantly earlier than females, and prenatal drug treatment having no significant effect on this reflex (Figure S3D). Negative geotaxis was monitored daily on P7–10 to ensure motor reflex development. There was no delay among treatment groups and all animals showed improvement across days, *F*(3,588) = 3.66, *p* = 0.012 (Figure S3E). Lastly, on P15, a free-fall righting reflex test was performed. There was a significant Sex×Prenatal drug×Postnatal drug interaction, *F*(3,146) = 4.95, *p* = 0.003. Males receiving prenatal LPS and postnatal PPA had significantly higher scores on the free-fall righting reflex test than males receiving prenatal LPS and postnatal VEH, *p* = 0.002. Females receiving prenatal LPS and postnatal PPA had significantly lower scores on the reflex test than females receiving prenatal LPS and postnatal VEH (*p* = 0.031) and lower scores than females receiving prenatal 2VEH and postnatal PPA (*p* = 0.029) or prenatal PPA and postnatal PPA (*p* = 0.042, Figure 3B).

### Behavioral Tests in Adolescence

#### Open-Field locomotor activity

**Figure 4:**
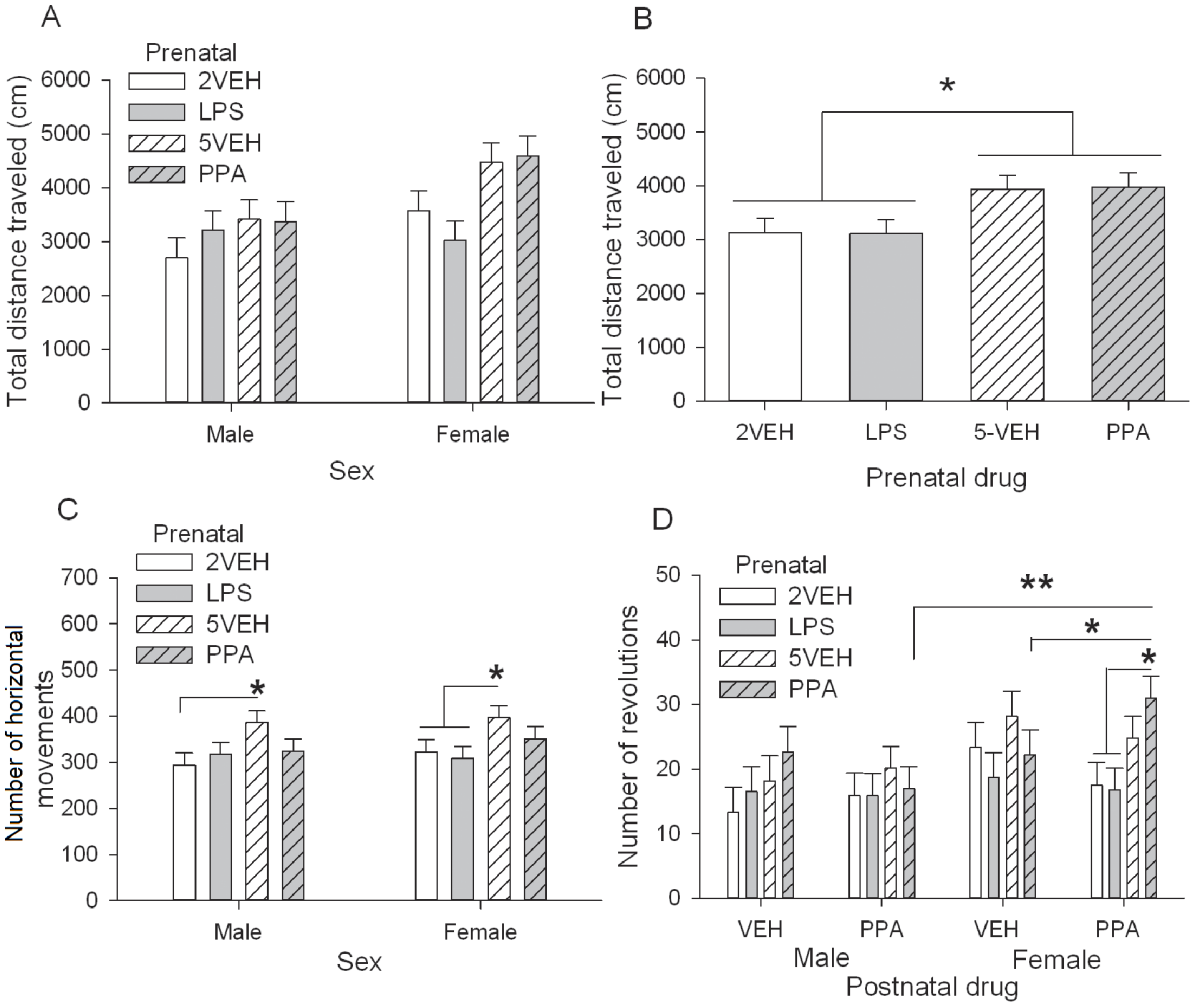
Locomotor activity in a novel open-field (P42) in male and female offspring. Adolescent females moved significantly more than males. A–B Total distance traveled (cm). Prenatal PPA and 5VEH treated animals moved significantly more than prenatal LPS and 2VEH treated animals, regardless of sex. C: Number of horizontal movements. An effect of prenatal drug showed animals in the prenatal 5VEH group made significantly more horizontal movements than the other 3 prenatal treatment groups. D: Number of revolutions. Females made significantly more revolutions than males. A double hit of prenatal PPA and postnatal PPA produced significantly more revolutions in female offspring compared to prenatal PPA alone in females and a double hit of PPA in males. Error bars represent S.E.M. Refer to Table 1 for group designations and sample sizes. * *p*<0.05, ** *p*<0.01.

Generally, regardless of drug treatment, female offspring were more active than male offspring for total distance traveled, *F*(1,100) = 8.55, *p = *0.004, horizontal movement time, *F*(1,100) = 6.31, *p = *0.014, and number of revolutions, *F*(1,93) = 11.86, *p = *0.001). Significant main effects of Prenatal drug were found for total distance traveled, *F*(3,100) = 3.23, *p* = 0.026, and number of horizontal movements, *F*(3,100) = 4.37, *p* = 0.006, but not for horizontal movement time. Animals prenatally exposed to PPA or 5VEH moved a significantly greater total distance than animals in both the LPS and 2VEH control group, *p*s <0.05 (Figure 4A–B). Further analysis showed this difference to be present in only female offspring as prenatal PPA and 5VEH treated females were significantly more active than prenatal LPS treated females, *p*s <0.01. Animals prenatally exposed to 5VEH performed a significantly greater number of horizontal movements than the other 3 prenatal drug groups (PPA *p* = 0.040, 2VEH *p* = 0.002, LPS *p* = 0.003), with the same effect in both male (5VEH significantly greater than 2VEH *p* = 0.014) and female offspring (5VEH significantly greater than 2VEH, LPS *p*s <0.05, Figure 4C).

There were no effects of prenatal LPS or PPA, or postnatal PPA treatment on vertical activity measures (number of vertical movements and vertical movement time). For number of revolutions, the Sex×Prenatal drug×Postnatal drug interaction was nearly significant, *F*(3,93) = 2.68, *p* = 0.051. Further analysis showed that a double hit of prenatal and postnatal PPA increased the number of revolutions made in the female offspring, but not in the male offspring (Figure 4D). Female offspring exposed to prenatal and postnatal PPA displayed significantly more revolutions than their male counterparts (*p* = 0.001), prenatal PPA-postnatal VEH treated females (*p* = 0.046), and female offspring exposed to prenatal LPS or 2VEH and postnatal PPA (*p*s <0.05).

Overall, prenatal PPA, prenatal LPS, and postnatal PPA alone did not produce hyper- or hypo-activity in male and female adolescent offspring. Prenatal PPA and postnatal PPA combined significantly increased the number of revolutions in female, not male offspring.

#### Open-Field thigmotaxis

**Figure 5:**
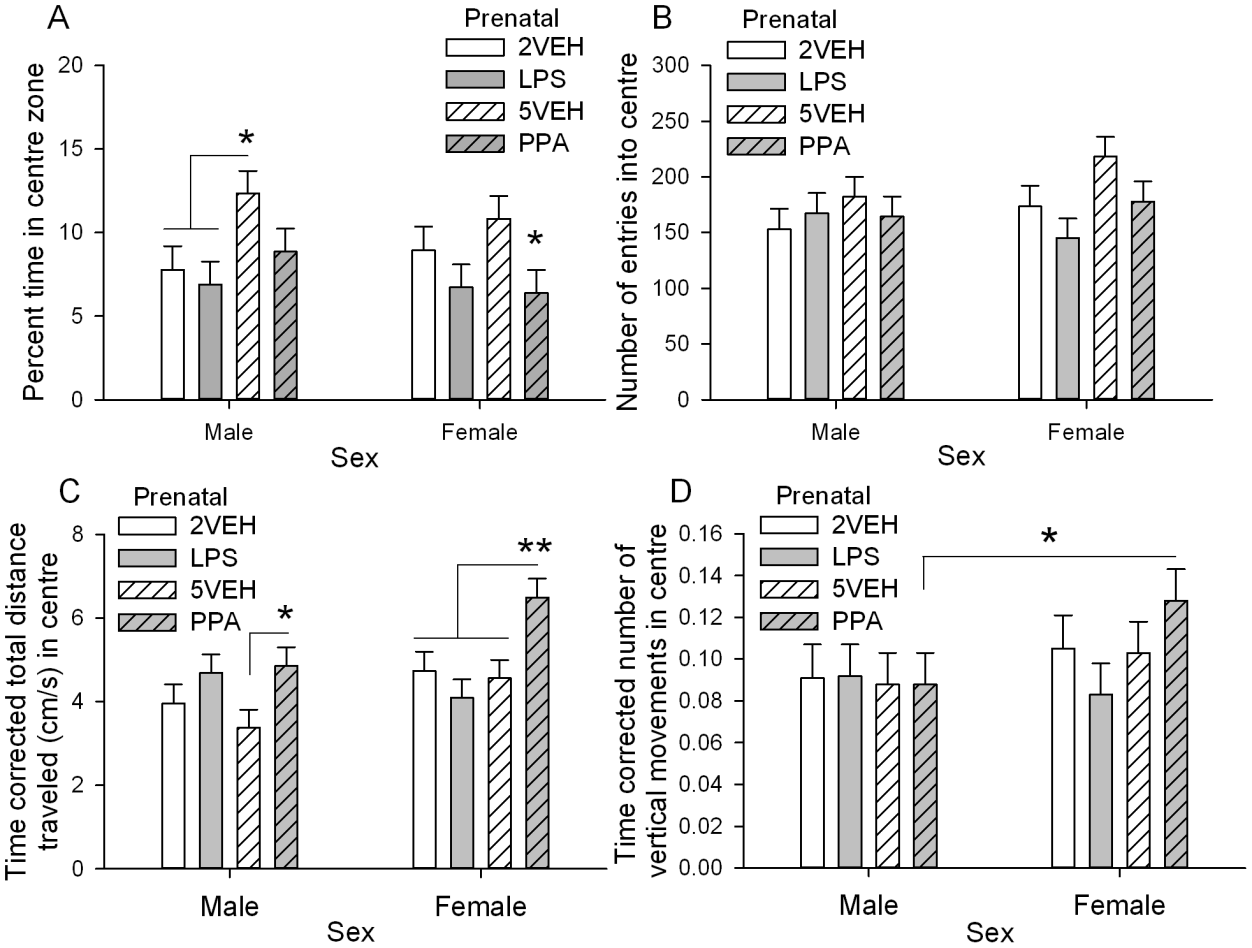
Thigmotaxis measures in a novel open-field (P42) in male and female offspring. Activity measures were time corrected. Females moved significantly more than males. A: Percent time in the centre of the open-field. Animals in the prenatal PPA treated group spent significantly less time in the centre of the open field than the prenatal 5VEH treated group. B: Number of entries into the centre. There were no significant differences in entries into the centre. C: Total distance traveled (cm/s) in the centre. Male and female offspring in the prenatal PPA treated group traveled significantly greater distances than the 5VEH treated control group. D: Number of vertical movements in the centre. Prenatal PPA treated female offspring reared significantly more than prenatal PPA treated male offspring. Error bars represent S.E.M. Refer to Table 1 for group designations and sample sizes. * *p*<0.05, ** *p*<0.01.

There was a significant main effect of Prenatal drug, *F*(3,100) = 4.83, *p* = 0.004, for percent time spent in the centre (Figure 5A). Prenatal PPA treated animals spent significantly less time in the centre of the open-field compared to prenatal 5VEH treated controls, *p* = 0.005. There was no significant difference between prenatal LPS and 2VEH animals. Prenatal 2VEH and LPS treated animals also spent significantly less time in the centre than animals treated with 5VEH (2VEH *p* = 0.023, LPS *p* = 0.001). There were no significant effects of treatment on the number of entries into the centre or perimeter of the open-field and postnatal PPA as compared to postnatal VEH had no significant effect on percent time in the centre or perimeter of the open-field (Figure 5B).

Locomotor activity measures were corrected for the amount of time spent in the perimeter or centre of the open-field. Prenatal LPS, prenatal PPA, and postnatal PPA did not significantly affect locomotor activity in the perimeter of the open-field on any horizontal or vertical activity measures (Figure S4A–D. Females traveled significantly greater total distances, Sex *F*(1,100) = 11.53, *p* = 0.001, and spent more time moving horizontally than males, Sex *F*(1,100) = 8.41, *p* = 0.005.

Postnatal PPA and prenatal LPS also did not affect central locomotor activity. However, prenatal PPA resulted in increased activity in the centre of the open-field. There were significant main effects of Sex, *F*(1,100) = 5.82, *p* = 0.018, and Prenatal drug, *F*(3,100) = 5.14, *p* = 0.002, for total distance traveled in the centre (Figure 5C) and a significant main effect of Prenatal drug, *F*(3,100) = 2.82, *p* = 0.043, for horizontal movement time in the centre (Figure S4E). Animals in the prenatal PPA group traveled significantly greater total distances than all other prenatal groups (5VEH *p*<0.001, LPS *p* = 0.006, 2VEH *p* = 0.008). This effect was present in both females (prenatal PPA significantly greater than other 3 groups, *p*s <0.01) and in males (prenatal PPA significantly greater than 5VEH, *p* = 0.021). For horizontal movement time, prenatal PPA treated animals spent significantly more time moving in the centre than 5VEH treated animals, *p* = 0.005.

While in the centre of the open-field, females generally performed more vertical movements, Sex *F*(1,93) = 6.56, *p* = 0.012, and spent more time engaged in rearing than males, Sex *F*(1,100) = 4.48, *p* = 0.037. A significant Sex×Prenatal drug interaction for number of vertical movements in the centre of the open-field, *F*(3,93) = 2.89, *p* = 0.040, indicated that females prenatally exposed to PPA performed a significantly greater number of vertical movements than prenatal PPA treated males, *p* = 0.001 (Figure 5D). No significant effects of prenatal treatments (LPS or PPA) were found for vertical time (Figure S4F).

Overall, prenatal LPS and postnatal PPA did not significantly alter time spent, or locomotor activity, in the perimeter or centre of the open-field. However, prenatal PPA significantly decreased time spent, and increased locomotor activity, in the centre of the open-field in both males and females.

#### Elevated Plus Maze

**Figure 6:**
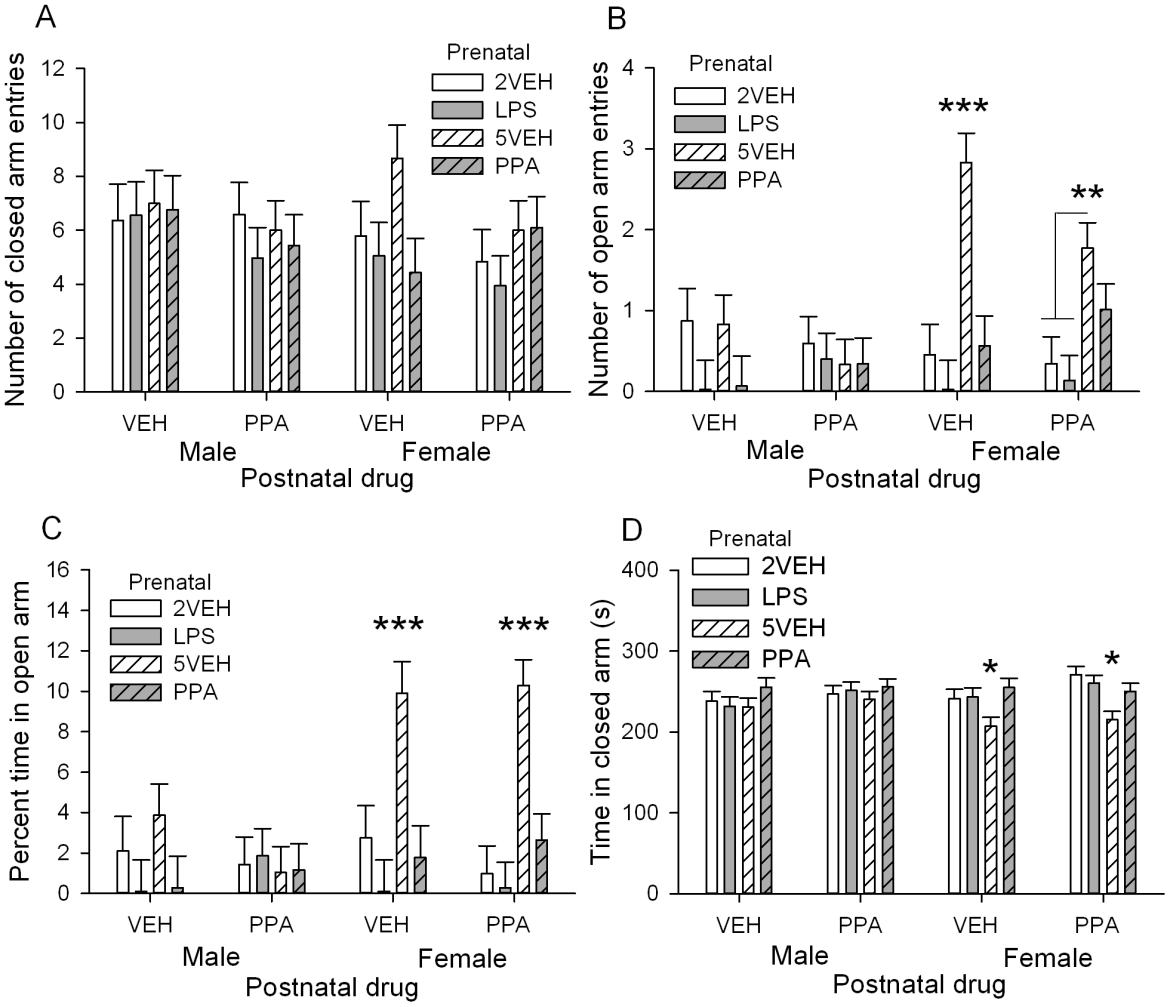
Elevated plus maze (P40) for male and female offspring. A: Number of closed arm entries was not significant. B: Number of open arm entries, C: Percent time in the open arm, D: Time in closed arm. Female offspring in the prenatal PPA treated group made less open arm entries, spent less time in the open arm, and spent more time in the closed arm than the prenatal 5VEH treated group. Error bars represent S.E.M. Refer to Table 1 for group designations and sample sizes. * *p*<0.05, ** *p*<0.01, *** *p*<0.001.

The number of entries into the closed arm was used as a measure of locomotion. There were no significant differences among groups in closed arm entries (Figure 6A). Number of entries into the open arm, percent time spent in the open arm, and closed arm time were used as traditional measures of anxiety-like behavior.

For number of entries into the open arm, there was a Prenatal drug×Postnatal drug interaction, *F*(3,92) = 2.99, *p* = 0.035. Postnatal PPA treated animals in the prenatal 5VEH group entered the open arm significantly fewer times than postnatal VEH animals (*p* = 0.01). A significant Sex×Prenatal drug interaction for both open arm entries, *F*(3,92) = 9.45, *p*<0.001, and percent time in the open arm, *F*(3,99) = 7.14, *p*<0.001, showed there were no differences in open arm entries among male offspring. Female offspring prenatally exposed to PPA entered the open arm significantly less often (*p* = 0.001) and spent significantly less time in the open arm than prenatal 5VEH treated female offspring (*p*<0.001, Figure 6B, 6C). Prenatal LPS and 2VEH treated females were also significantly less than prenatal 5VEH treated females for open arm entries and percent time in the open arm (*p*s <0.001). The significant Sex×Prenatal drug interaction, *F*(3,92) = 4.10, *p* = 0.009, for time spent in the closed arm showed that female offspring in the prenatal 5VEH group spent significantly less time in the closed arm than female offspring in the other 3 prenatal groups (*p*s <0.01, Figure 6D). Postnatal PPA treated animals also spent significantly more time in the closed arm than postnatal VEH treated animals, Postnatal drug *F*(1,92) = 6.74, *p* = 0.011, but this was only in female offspring (*p* = 0.037).

In summary, females in the prenatal PPA group made significantly less open arm entries, and spent less percent time in the open arm with more time in the closed arm than controls. Postnatal PPA significantly increased closed arm time in female offspring.

## Discussion

Until relatively recently, limited attention has been given to possible developmental effects of microbiome associated, GI metabolic products in rodents. Likewise, investigations of the effects of prenatal and postnatal immune activation with LPS and poly I:C have been mostly limited to adult male rodents. The present study investigated the effects of prenatal PPA and prenatal LPS in the emergence of postnatal developmental milestones, locomotor activity and on anxiety-like behavior in both male and female adolescent offspring. Additionally, a PPA regimen in the second week of life was used to evaluate if a subsequent postnatal insult would exacerbate any behavioral effects of prenatal treatment.

Prenatal LPS and PPA treatment resulted in developmental delays in male and female offspring, suggesting altered neurodevelopmental effects. Prenatal PPA alone did not influence open-field activity, but as expected, prenatal PPA produced increased anxiety-like behavior as evidenced by decreased time spent in the centre of the open-field in male and female adolescent rats, and less time on the open arm of the elevated plus maze (EPM) in females. Prenatal LPS did not alter behavior in the open-field and EPM. Postnatal PPA alone did not alter open-field activity, yet increased anxiety-like behavior in the EPM. Evidence for the double hit hypothesis was present in female adolescent rats, with the combination of PPA treatments increasing repetitive behavior, while no effect of a prenatal LPS and postnatal PPA double insult was observed. A double hit of prenatal LPS and postnatal LPS was beyond the scope of this study, but given evidence supporting two hits of LPS (postnatal and adolescence, [26]), this combination may provide important information and should be investigated in the future. Nonetheless, as a whole, the present results provide evidence that prenatal LPS or PPA and postnatal PPA can alter neurodevelopmental processes and that these changes manifest as sex- and test-specific alterations in activity and anxiety-like behavior in adolescent rats.

### Prenatal and Postnatal Treatments Produce Developmental Delay

Prenatal and postnatal treatments influenced body weight and developmental milestones. Body weight in male and female offspring was affected slightly by postnatal PPA treatment administered in the second week of life. Male pups were heavier and female pups were lighter than control pups, with the effects in males reaching significance across days. PPA administered in the first week of life and postnatal VPA in prior studies had no effect on body weight [23], [41]. However, while postnatal LPS has been demonstrated to not influence body weight in the first 3 weeks of life, there is some evidence of increasing body weight in males throughout adolescence [42], [43]. There were no differences in females with postnatal PPA and differences in males only in the prenatal 2VEH group at adolescence, suggesting minimal long-lasting effects on body weight.

Motor reflexes and development also developed normally in early life, consistent with previous studies in rats receiving LPS and VPA [36], [44]. In female offspring, there was a deficit in the ability to right mid-air on P15 in animals receiving a combination of prenatal LPS and postnatal PPA compared to prenatal vehicle treated controls. Prenatal VPA impaired free-fall righting in male and female offspring [45], while daily postnatal administration of PPA impaired free-fall righting in male offspring [23]. However, in males, prenatal LPS males receiving postnatal PPA were found to have higher righting ability scores compared to prenatal LPS-postnatal VEH treated males. These findings suggest that changes in activity in prenatal LPS and PPA treated adolescents are not due to developmental impairment in motor functions, but rather are associated with developmental changes in underlying neurobiological processes.

Physical developmental milestones monitored were normal in prenatal PPA and LPS treated pups compared to vehicle controls, with the exception of eye opening, which was delayed in both prenatal PPA and LPS treated male and female pups. These results are consistent with previous studies showing VPA delayed eye opening [22], [36]. In contrast, prenatal LPS on G15–16 at a higher dose than this study found no delay in eye opening [44], which may suggest that Long-Evans rats are more susceptible to developmental delay with prenatal LPS than Sprague-Dawley rats. Eye opening has been shown to be important for initiating glutamatergic synapse maturation [46]. As such, prenatal LPS and PPA treatment and postnatal PPA appear to have the capacity to affect some developmental processes.

### Prenatal PPA and Postnatal PPA Combined Produces Repetitive Behavior in Female Offspring

Overall, female adolescent rats displayed greater levels of basal locomotor activity than males, consistent with what is seen in adults [47]. Previously, central administration of PPA directly into the brain ventricles of adult males increased locomotion and repetitive behavior [48], [49]. While these acute administrations are not directly comparable to prenatal effects, they do point to possible direct locomotory effects with prenatal PPA. However, prenatal PPA or postnatal PPA alone did not affect locomotor activity. In fact, a ‘double hit’ of PPA in female offspring was required to produce an increase in repetitive movement, as measured by number of revolutions. This is similar to the results of Schneider et al. who reported a single injection of prenatal VPA increased duration and number of stereotypic movements in adult female rats, but not male rats [50].

Prenatal VPA has been shown to produce hyperactivity in male and female juvenile rats (P22–28) and adolescent male rats in an open-field [36], [51]. The present findings suggest that PPA at the present dosage may not be as effective as VPA and/or exert its effects through different mechanisms [52]. MIA offspring challenged with dopamine and NMDA drugs show increased locomotor activity compared to MIA controls challenged with the drugs [29], [30]. It is possible that PPA also affected dopamine neurotransmission [53], and that changes are subtle and might only appear if animals were challenged with psychomimetic drugs at the time of testing.

General activity in a novel open-field was greater in prenatal PPA and 5VEH treated offspring compared to LPS and 2VEH treated offspring. Receiving five injections may have induced a mild stress response and been enough of a stressor to affect development. Prenatally stressed rats have been shown to be hyperactive in a novel open-field, as well as displaying anxiety-like behavior [54], [55]. Repeated injections can alter baseline levels of plasma corticosterone [56], [57] and prenatal stress can compromise the placental barrier, exposing developing animals to corticosterone [58]. This may have led to greater activity in a potentially stressful situation, in this case, a novel open-field.

### Prenatal LPS did not Influence Locomotor Activity and Anxiety-like Behavior

General activity in an open-field does not seem to be altered by MIA alone in adolescent or adult offspring, as observed in this and prior studies [25], [29], [30]. This does not necessarily indicate that there are no developmental changes in neural functioning. For example, Fortier et al. found no change in open-field activity with a low dose of prenatal LPS until adult rats were challenged with amphetamine [29]. Alternatively, the open-field used in this study may have been too small to detect aversiveness to the centre of the open-field in prenatal LPS-treated rats. In a study with a larger novel open-field, prenatal LPS induced decreases in locomotor activity and time spent in the centre [25].

The low dose of LPS (50 µg/kg) administered during mid-late gestation (G15–16) may help explain why there were no effects of prenatal LPS on anxiety measures in the open-field and EPM. Increased anxiety-like behavior in the EPM was observed in adolescent male rats prenatally exposed to LPS on G16 or G17 at higher doses (100 and 150 µg/kg), and in adult mice offspring exposed to a similar dose of LPS as this study, but earlier prenatally (G10), or postnatally [24], [25]. While prenatal LPS produced developmental delay in rats, it was not sufficient to alter anxiety-like behavior.

### Prenatal and Postnatal PPA Increased Anxiety-like Behavior

When the open field was divided into centre and perimeter zones, prenatal PPA treatment increased anxiety-like behavior in both male and female offspring. Reduced time in the centre is indicative of increased anxiety, as the open space acts as an aversive space. Adolescent males and females prenatally exposed to PPA were also hyper-active when in the centre of the open-field, which may suggest a level of aversiveness. Adult rats fed a carbohydrate-rich diet exhibited anxiety and elevated levels of SCFAs in the gut [15] while previous research with MIA report decreased time in the centre of an open-field in adolescent male and female offspring [25], [28]. Additionally, infection of germ-free mice with a Gram-negative enteric pathogen (*Campylobacter jejuni*) increased anxiety behavior and was associated with increased early gene expression in brain regions implicated in anxiety [59]. However PPA, unlike LPS and *C. jejuni*, is not a pathogen and may not alter developmental processes in the same way. It is possible that similar immune processes are activated (e.g., cytokine release) as central PPA induces an innate neuroinflammatory response in the brain [48]; however, it remains to be seen if PPA in development alters immune function in rat offspring.

There were sexually dimorphic effects of prenatal and postnatal PPA treatment, with female rats displaying increased anxiety-like behavior in the EPM. Compared to 5VEH treated control females, time in the open arm and open arm entries were decreased in prenatal PPA treated females and closed arm time was increased in prenatal PPA and postnatal PPA treated females. An increase in anxiety is consistent with previous reports of prenatal VPA and enteric infection producing anxiety-like behavior in male and female adult rat offspring [50], [60].

A sex difference was present in the 5VEH control group, with males exhibiting similar levels of behavior as prenatal PPA treated males and females, suggesting anxiety in these control males. Brief daily periods of maternal stress resulted in increased anxiety in male adolescent rats, but not females [61]. Mild stress associated with the control injections may have increased exposure to corticosterone and altered developmental processes in males, thus preventing an effect of prenatal PPA in males from being significant. Studies of prenatal stress have shown sex differences in neurogenesis, decreased levels of testosterone and increased levels of corticosterone in offspring that may account for alterations in behavior (reviewed in [54]).

Similar to prenatal 5VEH treated males, both males and females treated with LPS and 2VEH displayed low levels of activity on the EPM. There may have been a ceiling effect in the current study that prevented an effect of LPS from being evident. It appears that this effect may be specific to these animals as behavioral testing occurred in a paired fashion (2VEH and LPS animals tested on the same day, and 5VEH and PPA animals tested together), and prenatal 5VEH treated females performed as expected. It is unclear why 2VEH and LPS treated animals produced such low levels of maze exploration. It has been shown that exposure to additional stressors has been required before increased anxiety-like behavior on the EPM was observed in rats treated with postnatal LPS [26]. Additionally, EPM results are sensitive to multiple environmental factors including prior housing condition, illumination levels, and prior handling (reviewed in [62]). Some aspect of the current EPM set-up may have induced a stressful state that led to decreased levels of exploration in the maze. Future investigations under different conditions may provide insight on this issue and how it relates to results of previous studies.

An imbalance between excitation and inhibition in the brain has been implicated in autism and anxiety, with changes in GABA and possibly serotonin suggested to be critical. Modifications in GABAergic systems have been reported in various brain regions of patients with ASD [63], [64] and these systems may be vulnerable to environmental agents during development. SCFAs and VPA can cross the placenta via monocarboxylate transporters and gain access to the developing fetus [65], [66]. PPA and VPA can act as histone deacetylase inhibitors and induce changes in gene expression [67], [68] with preliminary results demonstrating that central administration of PPA can alter gene expression in ASD associated genes (unpublished observations). Oral PPA during gestation and early life depleted whole brain GABA, serotonin, and dopamine and increased IL-6 in young rat brains [69]. As central administration of PPA has been found to induce an innate neuroinflammatory response [48], it is also possible that PPA in early life alters development through activation of immune processes. Placental immune responses to environmental agents may be sex-dependent [70]. Further investigation into possible direct and indirect mechanisms associated with PPA-induced alterations in development and behavior is needed.

While no behavioral effects of LPS were found in the current study, acute and chronic LPS during gestation induces a proinflammatory response and alters the placental barrier, which may allow external agents and/or cytokines to gain access to the fetus [71], [72]. Increases in proinflammatory cytokines, and changes in gene expression and GABAergic neurons have been found following prenatal LPS treatment [73], [74]. Finally, the current study assessed the behavioral effects of PPA and LPS in isolation. Evidence suggests a role for an altered microbiome in neurodevelopmental disorders [7], [34], other SCFAs have been shown to produce behavior and brain pathology similar to PPA in rats (acetate: [48]; butyrate: [49]), and it is more likely that combinations of many enteric metabolites, including PPA and LPS, contribute to the phenotypes observed in the human population, and may more accurately model the sex difference in these disorders.

## Conclusion

In summary, these results are the first to demonstrate that prenatal PPA, and one of a few to demonstrate that postnatal PPA and a low dose of LPS, alters developmental processes and subsequent behavior in male and female adolescent rats, resembling alterations observed in ASD and previous animal models. Prenatal LPS and prenatal PPA produced delay in eye opening and prenatal and postnatal PPA increased anxiety-like behavior in both male and female offspring, with a greater effect observed in female offspring. Developmental delay and altered temperament are observed in children with ASD, as reduced communication and motor skills or inappropriate emotional responses (e.g., passiveness) are observed [75] and anxiety disorders were the most common psychiatric conditions reported in ASD populations [2]. There was no male bias in PPA and LPS induced alterations in behavior, unlike the male predominance seen in ASD. A more balanced male to female ratio and the presence of gastrointestinal abnormalities was observed in children with ASD and mitochondrial disease (MD) [19]. It is possible that environmental insults contribute differently to the sex ratio observed in ASD. Additionally, evidence suggests that females with ASD display more severe behavioral symptoms than males and are likely to show more repetitive interests [76], [77]. The current results support this with a female sensitivity to PPA effects on repetitive behavior and anxiety. These results provide evidence that by-products of enteric bacteria metabolism can alter development and behavior in rats resembling that of ASD. Repeated infection or immune insult throughout gestation and early life may induce intestinal inflammation and alter the composition of the gut microbiome. Subsequent production of metabolic products, such as LPS and PPA, has the potential to adversely alter neurodevelopment in susceptible populations.

## Supporting Information

Figure S1 **Body weight (g) for male and female offspring from postnatal days 0–9.** A–B: Males. C–D: Females. Rats were prenatally exposed to either lipopolysaccharide (LPS) on G15–16, propionic acid (PPA) on G12–16, or their respective phosphate buffered saline controls (2VEH and 5VEH). There were no significant differences between prenatal treatment groups in body weight at birth or over the first 9 days of life. Error bars represent S.E.M. Refer to Table 1 for group designations and sample sizes.
(TIF)

Figure S2 **Body weight (g) for adolescent male and female offspring (P39–51).** A–B Males. C–D Females. Rats were prenatally exposed to either lipopolysaccharide (LPS) on G15–16, propionic acid (PPA) on G12–16, or their respective phosphate buffered saline controls (2VEH and 5VEH). Postnatal drug treatment, either PPA or VEH, was administered 2x/day every other day from P10–18. Males weighed significantly more than females (general effect, *p*<0.001), while males receiving postnatal PPA weighed significantly more than postnatal VEH treated males in the prenatal 2VEH group (2VEH-VEH vs. 2VEH-PPA in Panel A), * *p*s <0.05. Error bars represent S.E.M. Refer to Table 1 for sample sizes.
(TIF)

Figure S3 **Developmental milestones and reflexes for male and female offspring.** A: Pinna detachment. B: Top incisor eruption. C: Bottom incisor eruption. D: Righting reflex. Males performed the righting reflex slightly earlier than females (general effect, *p* = 0.011). E. Negative geotaxis. There were no significant differences between prenatal treatment groups in emergence of milestones and reflexes. Error bars represent S.E.M. Refer to Table 1 for group designations and sample sizes.
(TIF)

Figure S4 **Additional thigmotaxis measures (P42) in male and female offspring.** Activity measures were time corrected. A: Total distance traveled in the perimeter (cm/s). B: Horizontal movement time in the perimeter (s). Females were significantly greater than males for both total distance and movement time, *p*s <0.01. C: Number of vertical movements in the perimeter. D: Vertical time in the perimeter. There were no significant differences in vertical measures. E. Horizontal movement time in the centre (s). Prenatal PPA treated females spent significantly more time moving than prenatal 5VEH treated females. F: Vertical time (s) in the centre. Error bars represent S.E.M. Refer to Table 1 for group designations and sample sizes. * *p*<0.05.
(TIF)
