# Supplementary figures and images for: Pre- and Neonatal Exposure to Lipopolysaccharide or the Enteric Metabolite, Propionic Acid, Alters Development and Behavior in Adolescent Rats in a Sexually Dimorphic Manner
Source: PLoS One. 2014 Jan 22;9(1):e87072. doi: 10.1371/journal.pone.0087072 (PMC3899377; doi:10.1371/journal.pone.0087072)

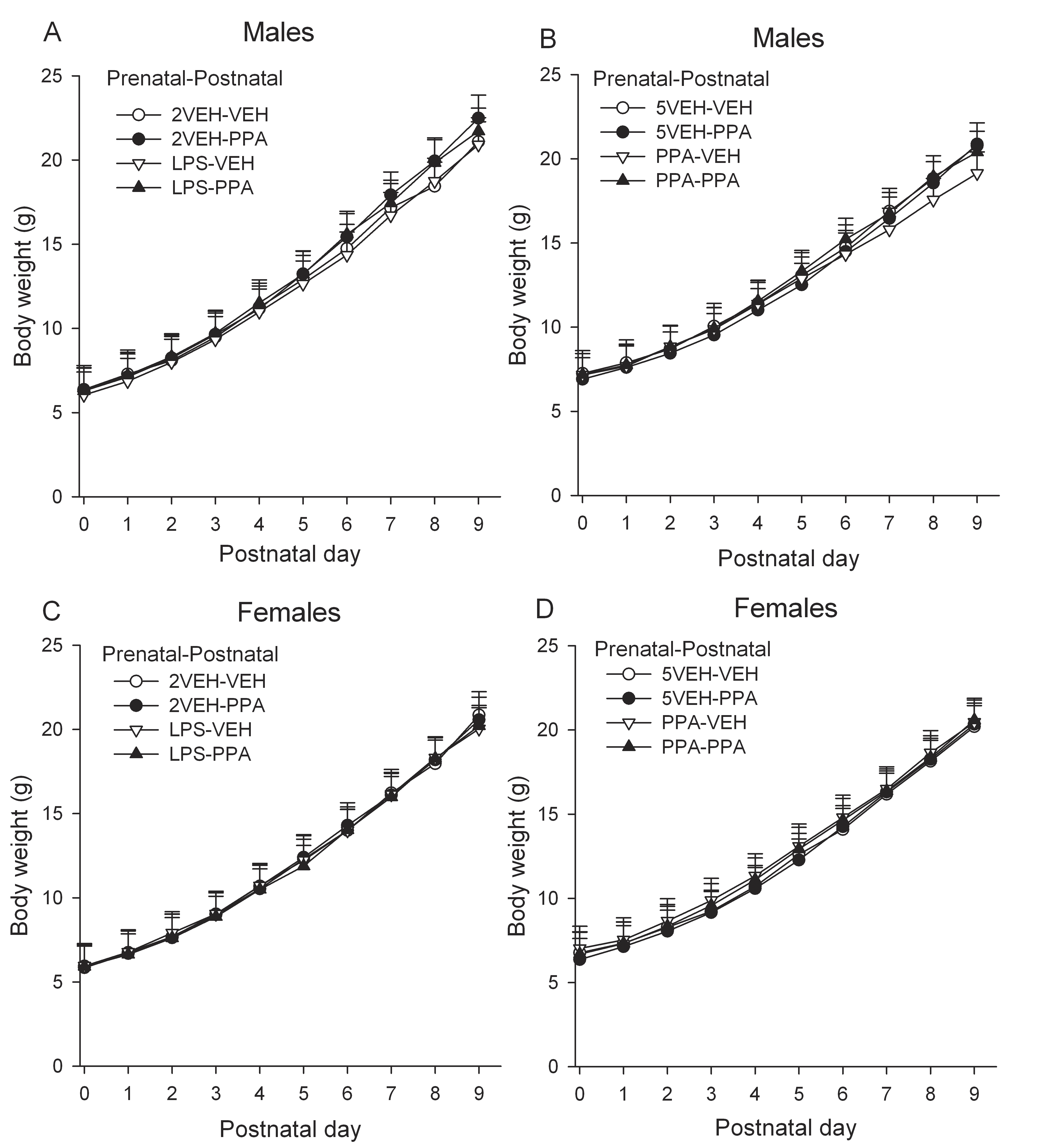

Supplement: Figure S1 — Body weight (g) for male and female offspring from postnatal days 0–9. A–B: Males. C–D: Females. Rats were prenatally exposed to either lipopolysaccharide (LPS) on G15–16, propionic acid (PPA) on G12–16, or their respective phosphate buffered saline controls (2VEH and 5VEH). There were no significant differences between prenatal treatment groups in body weight at birth or over the first 9 days of life. Error bars represent S.E.M. Refer to Table 1 for group designations and sample sizes. (TIF) [file pone.0087072.s001.tif]

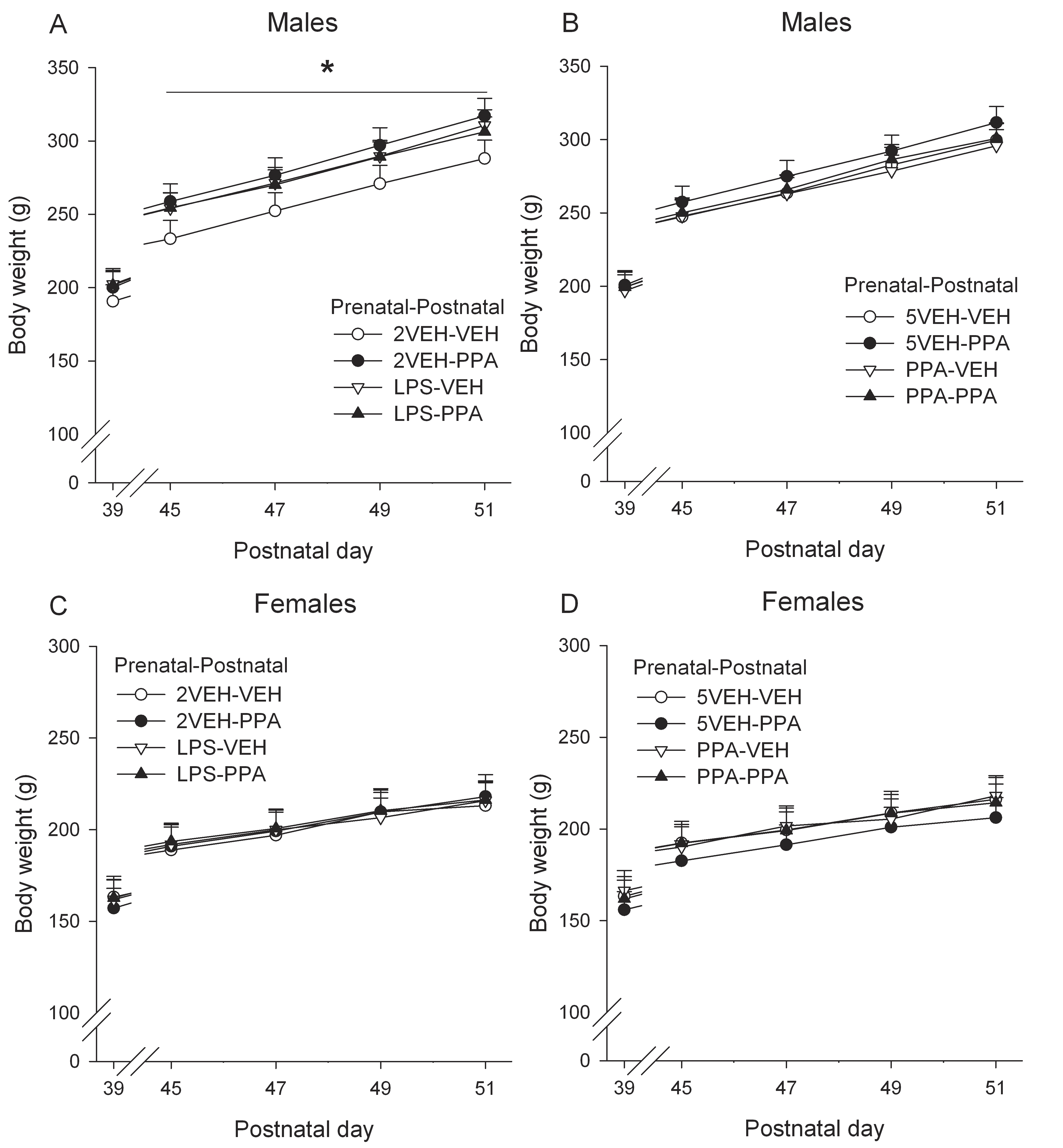

Supplement: Figure S2 — Body weight (g) for adolescent male and female offspring (P39–51). A–B Males. C–D Females. Rats were prenatally exposed to either lipopolysaccharide (LPS) on G15–16, propionic acid (PPA) on G12–16, or their respective phosphate buffered saline controls (2VEH and 5VEH). Postnatal drug treatment, either PPA or VEH, was administered 2x/day every other day from P10–18. Males weighed significantly more than females (general effect, p<0.001), while males receiving postnatal PPA weighed significantly more than postnatal VEH treated males in the prenatal 2VEH group (2VEH-VEH vs. 2VEH-PPA in Panel A), * ps <0.05. Error bars represent S.E.M. Refer to Table 1 for sample sizes. (TIF) [file pone.0087072.s002.tif]

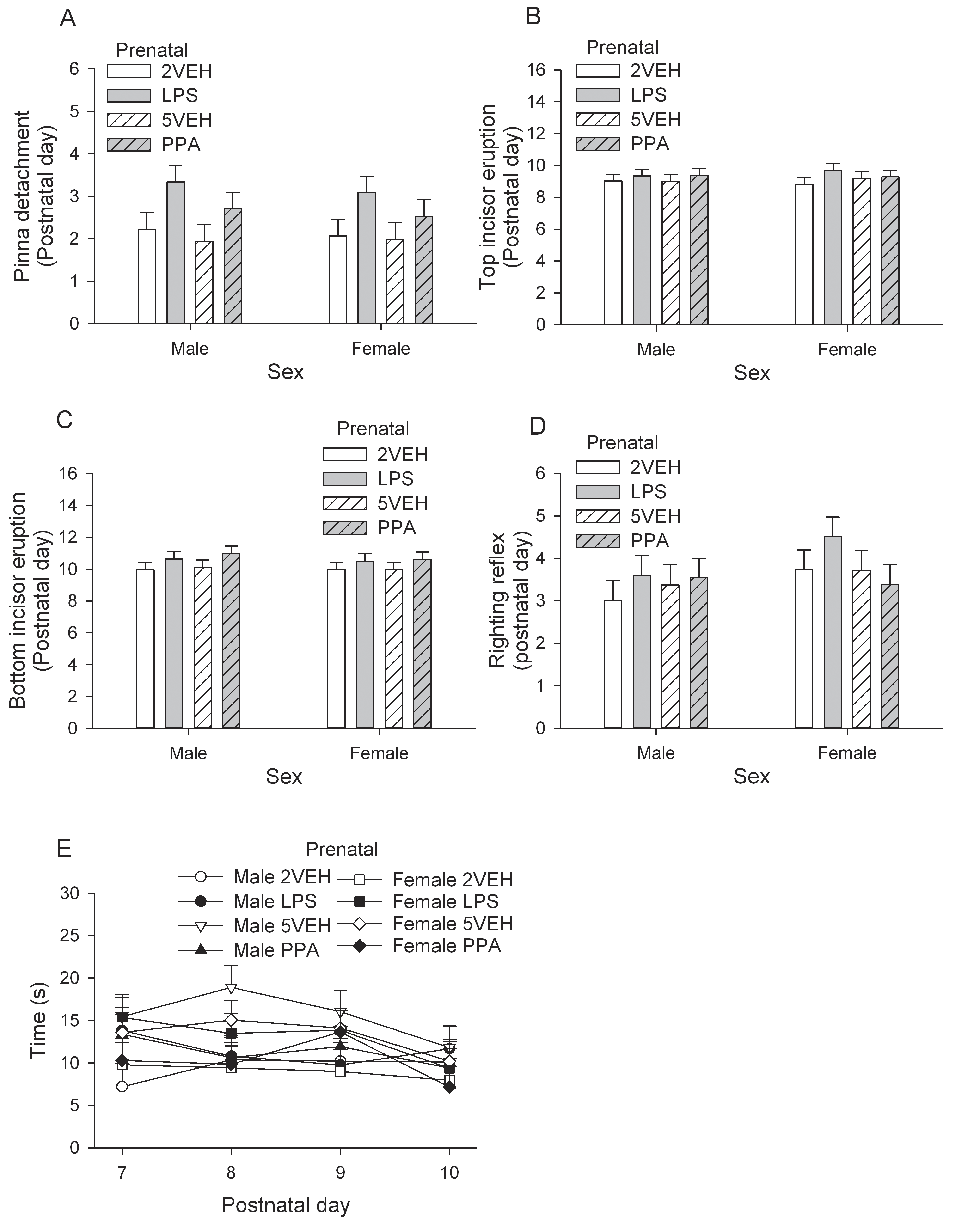

Supplement: Figure S3 — Developmental milestones and reflexes for male and female offspring. A: Pinna detachment. B: Top incisor eruption. C: Bottom incisor eruption. D: Righting reflex. Males performed the righting reflex slightly earlier than females (general effect, p = 0.011). E. Negative geotaxis. There were no significant differences between prenatal treatment groups in emergence of milestones and reflexes. Error bars represent S.E.M. Refer to Table 1 for group designations and sample sizes. (TIF) [file pone.0087072.s003.tif]

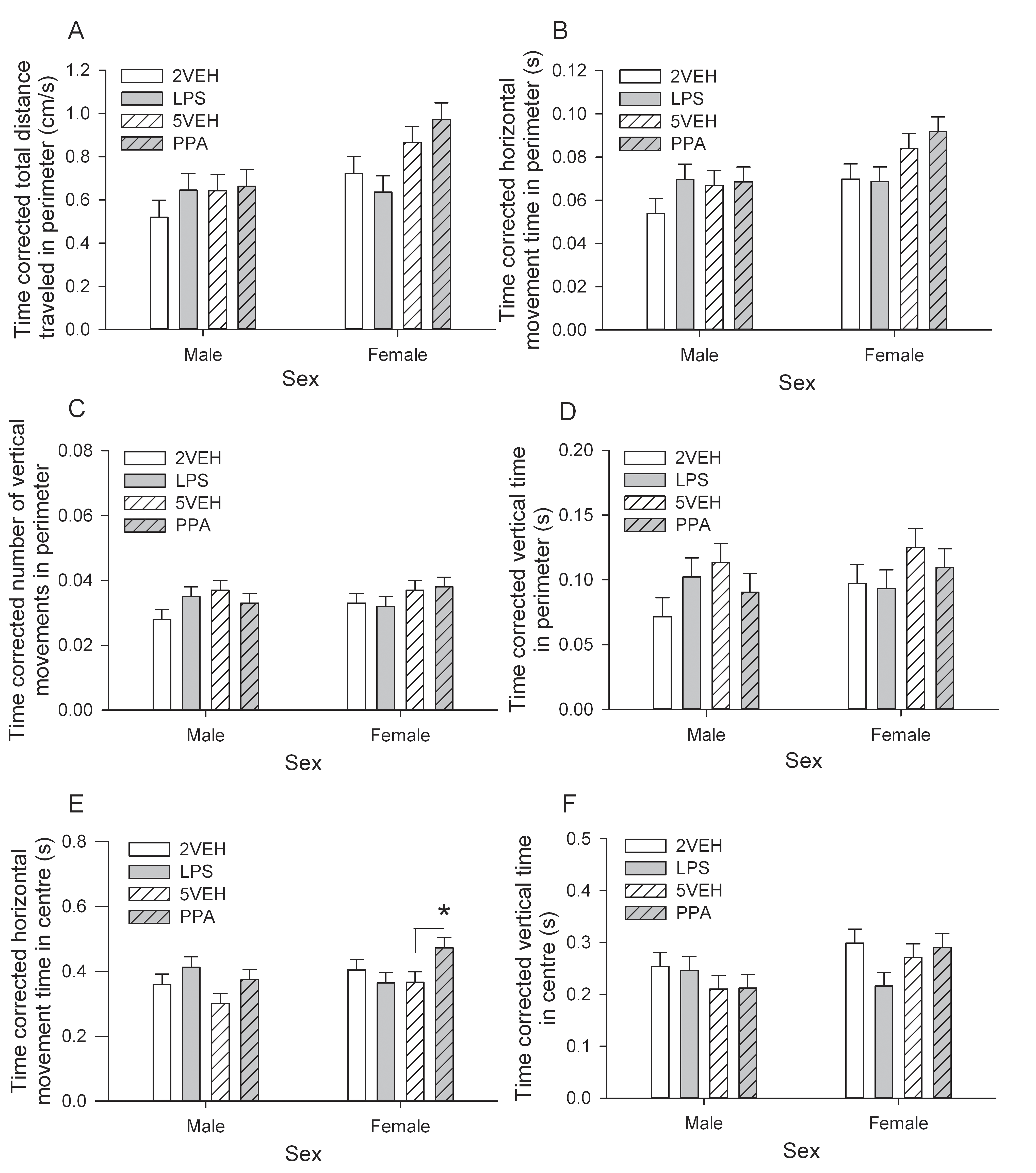

Supplement: Figure S4 — Additional thigmotaxis measures (P42) in male and female offspring. Activity measures were time corrected. A: Total distance traveled in the perimeter (cm/s). B: Horizontal movement time in the perimeter (s). Females were significantly greater than males for both total distance and movement time, ps <0.01. C: Number of vertical movements in the perimeter. D: Vertical time in the perimeter. There were no significant differences in vertical measures. E. Horizontal movement time in the centre (s). Prenatal PPA treated females spent significantly more time moving than prenatal 5VEH treated females. F: Vertical time (s) in the centre. Error bars represent S.E.M. Refer to Table 1 for group designations and sample sizes. * p<0.05. (TIF) [file pone.0087072.s004.tif]
